# Integrated Network Pharmacology, Molecular Docking and Experimental Validation Reveal That Quercetin Suppresses Clear Cell Renal Cell Carcinoma via MMP9-Associated Macrophage Polarization

**DOI:** 10.3390/biomedicines14040904

**Published:** 2026-04-16

**Authors:** Jinjing Huang, Dapeng Wang, Chengyun Xu, Jianping Wu

**Affiliations:** 1 Department of Nephrology, Second Affiliated Hospital, Nanchang University, 1 Minde Road, Donghu District, Nanchang 330008, China; h445922212@163.com; 2 Department of Neurosurgery, Center of Pituitary Tumor, Ruijin Hospital, Shanghai Jiaotong University School of Medicine, Shanghai 200025, China; wdpboj@126.com

**Keywords:** ***Cuscuta chinensis Lam.***, network pharmacology, molecular docking, clear cell renal cell carcinoma, immunomodulation, M2 macrophage

## Abstract

**Background**: Dodder, the dried mature seed of ***Cuscuta chinensis Lam.* (*CCL*)**, has demonstrated anti-tumor activity, but its molecular and immunological mechanisms in clear cell renal cell carcinoma (ccRCC) remain unclear. **Objective**: To identify potential targets and elucidate the immune mechanisms by which **CCL** exerts therapeutic effects against ccRCC. **Methods**: A network pharmacology approach was employed to predict **CCL**’s bioactive components and their putative targets in ccRCC. Gene Ontology (GO) and Kyoto Encyclopedia of Genes and Genomes (KEGG) enrichment analyses were used to explore relevant pathways. Molecular docking validated the binding of key compounds to hub proteins. In vitro assays—including cell viability, colony formation, invasion, and apoptosis measurements—assessed the effects of quercetin, a principal **CCL** constituent, on 786-O renal carcinoma cells. Flow cytometry was performed to determine the percentage of CD163^+^ cells. An in vivo xenograft model evaluated **CCL**’s anti-tumor efficacy. Western blotting, flow cytometry, and multiplex immunohistochemistry (mIHC) examined the modulation of signaling pathways and immune cell markers. **Results**: Network pharmacology identified IL-6, EGFR, TLR4, MMP9, CD44, and IFN-γ as core targets of **CCL** in ccRCC. Enrichment analyses implicated immune regulation, inflammation modulation, and PI3K/AKT signaling inhibition. Molecular docking revealed strong quercetin–MMP9 binding affinity. Immuno-correlation analyses indicated that high MMP9 levels positively correlated with macrophage infiltration and M2 polarization, suggesting a role in tumor immune escape. Quercetin significantly reduced the viability of 786-O cells in a dose-dependent manner, showing approximately 45% inhibition at 80 μM (*p* < 0.01). In addition, quercetin decreased MMP9 expression and reduced the proportion of CD163-positive macrophages. These effects were reversed by FSL-1 TFA (Toll-like receptor 2/6 agonist), which is the agonist of MMP-9. In the xenograft model, tumor volume in the quercetin-treated group was reduced by approximately 50% compared with the control group. **Conclusions**: **CCL**, particularly its active component quercetin, may inhibit ccRCC progression via inhibiting MMP9-mediated M2 macrophage polarization.

## 1. Introduction

ccRCC is the most frequently occurring type of kidney malignancy, constituting nearly 90% of all renal malignancies [1,2,3]. It is characterized by its high metastatic potential and poor prognosis, making it a significant clinical challenge [4,5]. Despite advancements in conventional therapies such as surgery, radiation therapy, and chemotherapy, ccRCC often relapses or metastasizes, necessitating the exploration of novel therapeutic strategies [6,7,8,9]. In addition, current targeted therapies (sunitinib, pazopanib) and immunotherapies (immune checkpoint inhibitors) have improved outcomes for ccRCC patients [10]. However, their efficacy is often limited by primary or acquired resistance, as well as significant inter-individual variability. Moreover, these treatments may not fully address the complex tumor microenvironment and immune evasion mechanisms that drive ccRCC progression. Therefore, identifying alternative therapeutic approaches that can complement existing strategies or overcome their limitations is critically needed. TCM, with its multi-targeted and multi-pathway mechanisms, has long been recognized for its potential to offer alternative approaches to cancer treatment [11,12,13,14,15]. ***CCL***, a key herb in traditional Chinese medicine, is recognized for its diverse bioactive properties, such as antioxidant, anti-inflammatory, and anticancer effects [16,17,18,19]. Recent research indicates that **CCL** could be effective against ccRCC, although the molecular mechanisms involved are not well-understood. To bridge this knowledge gap, we utilized network pharmacology as a systems biology approach to identify potential therapeutic targets of **CCL** in ccRCC and to investigate its molecular mechanisms.

Macrophage polarization plays a crucial role in the tumor immune microenvironment [20]. Macrophages can differentiate into functionally distinct phenotypes in response to microenvironmental signals: classically activated (M1) macrophages exhibit pro-inflammatory and antitumor activities, while alternatively activated (M2) macrophages promote anti-inflammatory responses, tissue repair, and tumor progression [21]. Tumor-associated macrophages (TAMs) can be broadly divided into classically activated M1 macrophages with anti-tumor functions and alternatively activated M2 macrophages that promote tumor growth, immune suppression and extracellular matrix remodeling. Increasing evidence suggests that M2 macrophage polarization contributes to the progression of clear cell renal cell carcinoma (ccRCC). Therefore, targeting macrophage polarization may represent a promising therapeutic strategy.

To systematically investigate the potential mechanisms of **CCL** in ccRCC, we employed network pharmacology—a systems biology approach that provides a comprehensive framework for understanding complex interactions between natural compounds and biological targets [22,23]. By analyzing the bioactive components of **CCL** and their predicted interactions with key pathways and genes, we aim to identify potential therapeutic targets and pathways. Bioinformatics tools, including pathway enrichment analysis (KEGG and GO), protein–protein interaction (PPI) networks, and molecular docking, were utilized to predict the functional significance of **CCL**’s targets in ccRCC. These in silico approaches generate hypotheses regarding the molecular mechanisms by which **CCL** may exert its anticancer effects, with particular attention to immune modulation and M2 macrophage polarization. The overall workflow of this study is illustrated in the Graphical Abstract.

## 2. Materials and Methods

### 2.1. Chemicals and Reagents

RNAiso Plus was obtained from TaKaRa (Shiga, Japan). Quercetin (HY-18085A, purity 98.45%) and FSL-1 TFA (HY-P2036A, purity 99.81%) were purchased from MedChemExpress (Monmouth Junction, NJ, USA). The Cell Counting Kit-8 (CCK-8) was acquired from Dojindo (Kumamoto, Japan). The Annexin V-FITC/PI apoptosis detection kit was supplied by BD Biosciences (San Jose, CA, USA).

For Western blot analysis, the following primary antibodies were used: anti-MMP9 (1:1000 dilution; Abcam, Cambridge, MA, USA), anti-CDK4, anti-BAX, anti-BCL2, anti-PI3K, anti-AKT1 (all at 1:1000 dilution; Cell Signaling Technology, Danvers, MA, USA), and anti-GAPDH (1:2000 dilution; Cell Signaling Technology, Danvers, MA, USA).

For immunohistochemistry (IHC) and immunofluorescence (IF) analyses, the following primary antibodies were employed: anti-MMP9 (1:200 dilution; Abcam, Waltham, MA, USA), anti-CD163 (1:200 dilution; Abcam, Waltham, MA, USA), anti-CD80 (1:200 dilution; Cell Signaling Technology, Danvers, MA, USA), anti-Ki67 (1:800 dilution; Cell Signaling Technology, Danvers, MA, USA), and anti-Bax (1:100 dilution; Abcam, Waltham, MA USA). Additionally, anti-human CD163 antibody (clone: GHI/61, catalog: 556018) and its corresponding isotype control antibody (Mouse IgG1, catalog: 556027) were obtained from BD Biosciences (San Jose, CA, USA).

### 2.2. Identification of Bioactive Compounds and Target Proteins in **CCL**

Active compounds in **CCL** were identified using the Traditional Chinese Medicine Systems Pharmacology Database and Analysis Platform (TCMSP, https://www.tcmsp-e.com/molecule.php?qn=11865, accessed on 15 March 2025). The criteria for selection included an oral bioavailability of at least 30% and drug-likeness of 0.18 or higher. The identified compounds were further screened for their putative targets using the Swiss TargetPrediction and Comparative Toxicogenomics Database (CTD). The obtained targets were merged, and duplicate entries were removed. Gene standardization utilized the UniProt database (https://www.uniprot.org).

### 2.3. Identification of Potential Targets in ccRCC

Differentially expressed genes linked to ccRCC were obtained from the Gene Expression Omnibus (GEO) database (https://www.ncbi.nlm.nih.gov/geo/, accessed on 15 March 2025). The analysis of GSE20718 was conducted using the ‘limma’ package in R, applying thresholds of |logFC| > 1 and an adjusted *p*-value < 0.05. Visualization was performed using “ggplot2” and “pheatmap” for volcano plots and heatmaps. The overlapping genes between **CCL** targets and ccRCC-related DEGs were identified using VennDiagram 1.8.2, and these overlapping targets were considered as potential therapeutic targets.

### 2.4. Gene Function and Pathway Enrichment Analysis

Functional relevance of the identified targets was explored through GO and KEGG enrichment analyses. GO analysis evaluated their involvement in biological processes, molecular functions, and cellular components, while KEGG pathway analysis identified key signaling pathways in ccRCC. Analyses were conducted using the ‘clusterProfiler 4.18.1’ package in R, with results visualized through bar plots and bubble charts.

### 2.5. PPI Network Analysis

The analysis of protein–protein interaction (PPI) networks was conducted to elucidate the functional relationships between proteins. By constructing a PPI network, we aimed to identify key proteins and interactions that may play significant roles in biological processes. The network was analyzed using various computational tools to determine central nodes and clusters, providing insights into the underlying molecular mechanisms. A PPI network was constructed using the STRING database (https://string-db.org/) with a minimum confidence score of 0.9. The network was analyzed with Cytoscape 10.0 to identify hub genes using centrality algorithms such as Maximal Clique Centrality (MCC), Degree, and Edge Percolated Component (EPC).

### 2.6. Molecular Docking

The interaction between key bioactive compounds of **CCL** and target proteins was evaluated using molecular docking [24]. The 3D structures of the compounds were obtained from the PubChem database, while those of the target proteins were retrieved from the Protein Data Bank (PDB, https://www.rcsb.org/) including MMP9 (PDB ID: 1L6J), CD44 (PDB ID: 4PZ3), IL-6 (PDB ID: 1ALU), and TLR4 (PDB ID: 3FXI). AutoDockTools 1.5.6 was utilized for docking, and binding energy (kcal/mol) was used to assess interaction strength. PyMOL3.1.8 was utilized to visualize the docking results.

### 2.7. Cell Culture

The 786-O human renal carcinoma cell line, sourced from a certified cell bank, was cultured in RPMI-1640 medium supplemented with 10% fetal bovine serum and 1% penicillin-streptomycin, and incubated at 37 °C with 5% CO_2_ for optimal growth. The medium was refreshed bi-daily, and cells were subcultured at 80–90% confluency with 0.25% trypsin-EDTA. All experiments utilized cells in the logarithmic growth phase.

### 2.8. Cell Viability Assay

Cell viability was assessed using the Cell Counting Kit-8 (CCK-8, 40203ES, Shanghai, China) assay according to the manufacturer’s instructions [25]. A total of 5000 786-O cells per well were seeded in 96-well plates and allowed to adhere overnight. Cells were exposed to varying concentrations of quercetin (0, 20, 40, 80 μM) dissolved in DMSO to prepare a stock solution or FSL-1 TFA for 24 h. Following treatment, each well received 10 μL of CCK-8 reagent and was incubated at 37 °C for 2 h. Absorbance at 450 nm was recorded using a BioTek microplate reader (Molecular Devices, San Jose, CA, USA). Experiments were conducted in triplicate, with cell viability represented as a percentage relative to the control group.

### 2.9. Colony Formation Assay

The 786-O cells were seeded at 500 cells per well in 6-well plates and allowed to adhere overnight. Cells were exposed to quercetin at concentrations of 0, 20, 40, and 80 μM or quercetin +FSL-1 TFA for 14 days, with the culture medium refreshed every three days. Post-incubation, colonies were treated with 4% paraformaldehyde for 15 min and subsequently stained with 0.5% crystal violet solution for 20 min at room temperature. Colonies were stained, washed with PBS, air-dried, and counted using ImageJ software 1.8.0. Colonies with over 50 cells were deemed viable.

### 2.10. Transwell Assay [26]

Cell invasion was assessed using Transwell chambers (Corning, NY, USA) equipped with 8-μm pore size polycarbonate membranes, pre-coated with Matrigel (BD Biosciences, USA) at a 1:8 dilution. The 786-O cells (1 × 10^5^ cells/well) were seeded in 200 μL of serum-free medium in the upper chamber, with 600 μL of RPMI-1640 medium containing 10% FBS in the lower chamber. After a 24-h exposure to quercetin at 0, 20, 40, and 80 μM, or quercetin +FSL-1 TFA, non-invading cells were removed from the upper membrane using a cotton swab. Invaded cells were fixed with 4% paraformaldehyde, stained with 0.1% crystal violet, and examined under an Olympus light microscope. Invasion ability was assessed by counting cells in five random fields per chamber.

### 2.11. Apoptosis Assay

The Annexin V-FITC/PI apoptosis detection kit was used to analyze apoptotic cell death, which was evaluated through flow cytometry [27]. The 786-O cells (1 × 10^6^ per well) were cultured in 6-well plates and exposed to quercetin at concentrations of 0, 20, 40, and 80 μM for 24 h. After treatment, cells were harvested by trypsinization without EDTA, washed twice with cold PBS, and resuspended in 100 μL of 1× binding buffer. Cells were incubated with 5 μL Annexin V-FITC and 5 μL PI for 15 min in the dark at room temperature. Apoptosis rates were assessed via a BD FACSCanto II flow cytometer (BD Biosciences, San Jose, CA, USA) and analyzed using FlowJo software V11.1. Cells in early apoptosis were marked as Annexin V+/PI−, while those in late apoptosis were identified as Annexin V+/PI+.

### 2.12. RT-qPCR

Total RNA was extracted from 786-O cells using RNAiso Plus, followed by reverse transcription with the PrimeScript™ RT Reagent Kit (Takara Bio Inc., Shiga, Japan). Amplification utilized the TBBR-PremixExTaq™ Kit (Takara Bio Inc., Shiga, Japan), and detection occurred on a CFX96 Touch™ (Bio-Rad Laboratories, Inc., Hercules, CA, USA) Real-Time System. The target gene’s relative expression was determined using the 2^−ΔΔCt^ method and was normalized against the reference gene GAPDH. The primer sequences used in this study were as follows: GAPDH, Forward: 5′-GTCAAGGCTGAGAACGGGAA-3′, Reverse: 5′-AAATGAGCCCCAGCCTTCTC-3′; CD163, Forward: 5′-GGAGTTGCCCTTTCTACCCC-3′, Reverse: 5′-TACCAGGCGAAGTTGACCAC-3′; CD206, Forward: 5′-GGAGTTGCCCTTTCTACCCC-3′, Reverse: 5′-CTGTCCGCCCAGTATCCATC-3′; CD86, Forward: 5′-CAGGGACTAGCACAGACACAC-3′, Reverse: 5′-CAGGTTGACTGAAGTTAGCAGAG-3′.

### 2.13. Western Blot Analysis

Protein extraction from the 786-O cells was performed using RIPA lysis buffer (Beyotime, Shanghai, China) supplemented with protease and phosphatase inhibitors. The protein concentration was measured using the BCA protein assay kit (Thermo Fisher, Waltham, MA, USA). Proteins, each weighing 30 μg per sample, were separated using 10% SDS-PAGE and then transferred to PVDF membranes from Millipore (Burlington, MA, USA). Membranes were blocked with 5% non-fat milk in TBST for 1 h at room temperature, followed by overnight incubation at 4 °C with primary antibodies. The membranes were washed thrice with TBST and incubated with HRP-conjugated secondary antibodies (1:5000, CST, Danvers, MA, USA) for one hour at room temperature. Protein bands were detected with enhanced chemiluminescence (ECL) reagent (Thermo Fisher, USA) and captured using a ChemiDoc XRS+ imaging system (Bio-Rad, Hercules, CA, USA). Protein expression levels were quantified using ImageJ software, employing GAPDH as the internal reference.

### 2.14. Xenograft Mouse Model

A xenograft model using BALB/c nude mice was created by subcutaneous injection of 1 million 786-O cells into each mouse. Two groups of mice were formed randomly, including a control group that received vehicle treatment and a quercetin-treated group that was administered 50 mg/kg/day quercetin orally for 18 days. Tumor growth was monitored by measuring volumes every three days using the formula: Tumor volume = (length × width^2^)/2. Tumors were excised, weighed, and subjected to histological and molecular analysis at the study’s conclusion.

### 2.15. mIHC Staining

To analyze the co-expression of MMP9, CD163, and CD80, mIHC was performed using tissue sections from xenograft tumors. The sections underwent deparaffinization followed by heat-induced antigen retrieval in a pH 6. 0 citrate buffer at 95 °C for 15 min. To reduce background fluorescence, slides were blocked with 5% BSA for 1 h at room temperature. The sections were incubated overnight at 4 °C with a primary antibody mixture comprising. Following washing, the slides were incubated in the dark at room temperature for 1 h with fluorescently labeled secondary antibodies (Alexa Fluor 520, 570, and 650, Thermo Fisher, USA) at a 1:500 dilution. DAPI (1:1000, Thermo Fisher, USA) was used for nuclear staining for 10 min. Images were captured using a Leica TCS SP8 (Wetzlar, Germany) confocal laser scanning microscope, and fluorescence intensity was analyzed with ImageJ software.

### 2.16. Determination of the Percentage of CD163^+^ Cells

After the 24-h co-culture period under different treatment conditions (Control, quercetin, quercetin + DMSO, quercetin + FSL-1 TFA), cells were harvested and washed with cold phosphate-buffered saline (PBS). To detect CD163-positive cells, the cell pellet was resuspended in 100 µL of staining buffer (e.g., PBS containing 1% FBS) and incubated with 5 µL of a fluorescently conjugated BD Horizon™ anti-human CD163 antibody for 30 min in the dark at 4 °C. An equivalent amount of the corresponding BD isotype control antibody was used under identical conditions to establish background staining. Following incubation, the cells were washed twice with staining buffer to remove unbound antibodies and finally fixed in 1% paraformaldehyde (PFA) in PBS. Data acquisition was performed immediately on a flow cytometer (CytExpert SRT, Beckman, Indianapolis, IN, USA), and a minimum of 10,000 events per sample within the live cell gate were collected.

### 2.17. Statistical Analysis

Experiments were performed in triplicate, with results expressed as the mean ± standard deviation (SD). GraphPad Prism 9. 0 (GraphPad Software, San Diego, CA, USA) was used for statistical analysis. An unpaired, two-tailed Student’s *t*-test was used for comparing two groups, while one-way ANOVA with Tukey’s post hoc test was applied for multiple group comparisons. Pearson’s correlation coefficient (r) was used for the correlation analysis. Kaplan–Meier survival curves were analyzed using the log-rank (Mantel–Cox) test.

## 3. Results

### 3.1. Identification of Potential Targets of **CCL** in ccRCC Based on Network Pharmacology

**Figure 1:**
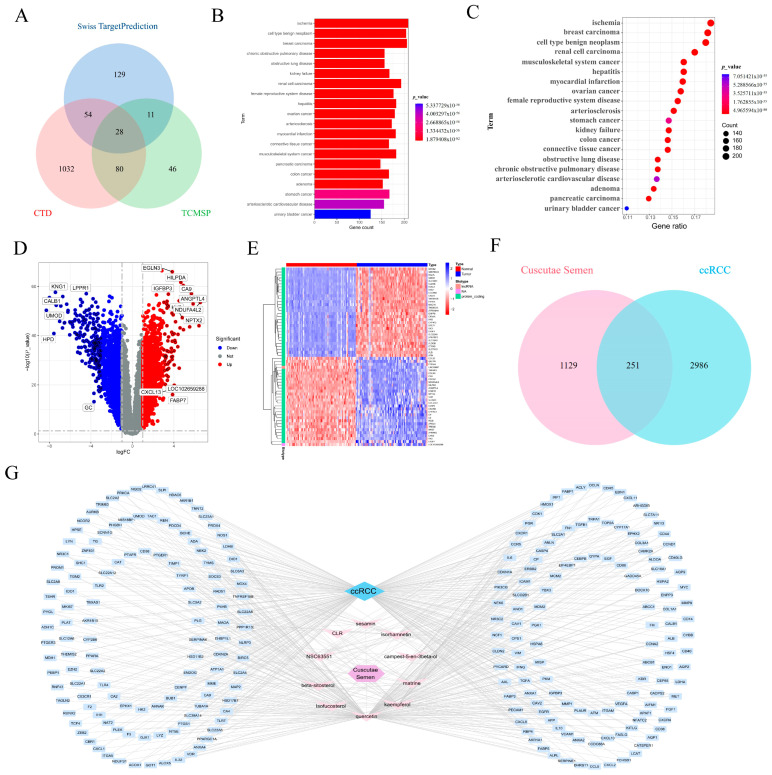
Identification of Potential Targets of **CCL** in ccRCC Based on Network Pharmacology. (**A**) VN diagram visualizes **CCL** targets in the Swiss Target Prediction, CTD and TCMSP databases. (**B**,**C**) DO analysis of disease enrichment corresponding to the top 20 **CCL** targets. (**D**) Volcano map visualization |logFC| ≥ 1 ccRCC differentially expressed gene. (**E**) The top 30 differentially expressed genes upregulated and downregulated in normal and tumor tissues in the heat map visualization GEO database. (**F**) VN diagram visualizes common potential targets of **CCL** and ccRCC. (**G**) Cytoscape visualization “**CCL**-Active Ingredient-Target-ccRCC” network construction.

Eleven active components of **CCL** were identified from the TCMSP database, and the screening information of active medicinal components is shown in Table 1, while 1379 related targets were gathered from the TCMSP, CTD, and Swiss TargetPrediction databases concurrently (Figure 1A). DO disease enrichment analysis was performed on these targets to obtain the first 20 items, mainly involving nephro-related diseases (Figure 1B,C). A volcano plot confirmed the differential expression of genes linked to ccRCC. The plot identifies genes that were significantly upregulated (in red) and downregulated (in blue), offering a clear visualization of the gene expression changes in ccRCC patients (Figure 1D). A heatmap was used to show the expression patterns of the identified genes across different conditions. The color gradient from red to blue indicates the variation in gene expression, revealing significant differences between normal and ccRCC tissues (Figure 1E). A Venn diagram comparing the overlapping genes between **CCL** and ccRCC revealed 251 shared targets, further strengthening the hypothesis that these genes play crucial roles in the therapeutic action of **CCL** against ccRCC (Figure 1F). Finally, the network of interactions between **CCL** and ccRCC-related genes was illustrated, showing how active compounds interact with the identified targets. The network analysis indicates possible therapeutic mechanisms by which **CCL** could influence ccRCC treatment (Figure 1G).

### 3.2. Analysis of Key Targets: Pathway and Functional Enrichment

Functional enrichment analysis was performed to investigate the biological significance of **CCL**’s potential targets in ccRCC. GO analysis revealed significant enrichment of these targets in biological processes related to immune response, inflammation, and hypoxia adaptation (Supplementary Figure S1A). KEGG pathway enrichment analysis identified key signaling pathways involved in ccRCC treatment. The most enriched pathways, such as lipid and atherosclerosis, PI3K-AKT signaling, HIF-1 signaling, and cytokine–cytokine receptor interaction, underscore their significance in cancer progression and immune modulation (Supplementary Figure S1B). The PI3K–AKT pathway, recognized for its functions in cell survival, proliferation, and immune regulation, was notably prominent, suggesting its potential role in the therapeutic effects of **CCL**. A PPI network was constructed to emphasize the primary interactions among target genes. The network identified several hub genes that play vital roles in crucial biological activities like signaling transduction, apoptosis, and cell cycle regulation, with highly interconnected nodes (Supplementary Figure S1C). The findings suggest that the therapeutic effects of **CCL** are likely mediated by intricate regulatory interactions among key signaling molecules. KEGG pathway analysis revealed that numerous identified targets were associated with the PI3K–AKT signaling pathway, with several genes showing significant upregulation or downregulation (Supplementary Figure S1D). This reinforces the hypothesis that **CCL** exerts its anti-ccRCC effects through modulating immune-related pathways and cancer progression mechanisms. These findings indicate that **CCL** therapeutically influences ccRCC by modulating immune responses, inflammation, and oncogenic pathways, notably via the PI3K–AKT signaling axis. Consistently, Western blot analysis confirmed that quercetin downregulated PI3K and AKT expression in a dose-dependent manner (Supplementary Figure S1E), further supporting the involvement of the PI3K–AKT axis in quercetin’s anti-tumor effect.

### 3.3. Network and Molecular Interactions of Key Genes in ccRCC

To further investigate the molecular mechanisms through which **CCL** exerts its effects in ccRCC, we performed a detailed analysis of the interactions between the identified key genes. The PPI network, depicted in Supplementary Figure S2A, was assembled. This network identified key genes such as IL6, FN1, CD44, TLR4, and EGFR, which were centrally positioned and potentially play significant roles in the therapeutic effects of **CCL** in ccRCC. The network also highlights other genes that are connected to immune regulation and cell migration, reinforcing the idea that these genes could mediate the therapeutic effects of **CCL** through immune modulation and metastasis inhibition. The PPI network analysis was extended to concentrate on the top 10 hub genes identified through various network analysis methods, including Maximal Clique Centrality (MCC), Degree, and Edge Percolated Component (EPC). In Supplementary Figure S2B, the MCC-based network identified IL6 and TLR4 as key nodes with the highest centrality, while Supplementary Figure S2C shows that the MNC (Maximum Neighborhood Component) analysis also emphasizes the importance of IL6and CD44. Supplementary Figure S2D presents the Degree-based network, revealing FN1 and CD44 as high-degree genes, indicating their central role in ccRCC progression. Supplementary Figure S2E, representing the EPC analysis, further validates the involvement of IL6, CD44, and MMP9 as key molecular markers. Together, these results point to the importance of IL6, CD44, and TLR4 in mediating the therapeutic effects of **CCL** in ccRCC. Finally, Supplementary Figure S2F shows a Venn diagram that highlights the overlapping genes from the three different network analysis methods (MCC, Degree, and EPC). This overlap emphasizes IL6, CD44, TLR4, MMP9, IFNG, and EGFE as key molecules involved in **CCL**’s therapeutic mechanism in ccRCC.

### 3.4. Molecular Docking Analysis of Active Compounds with Core Inflammatory Targets

**Figure 2:**
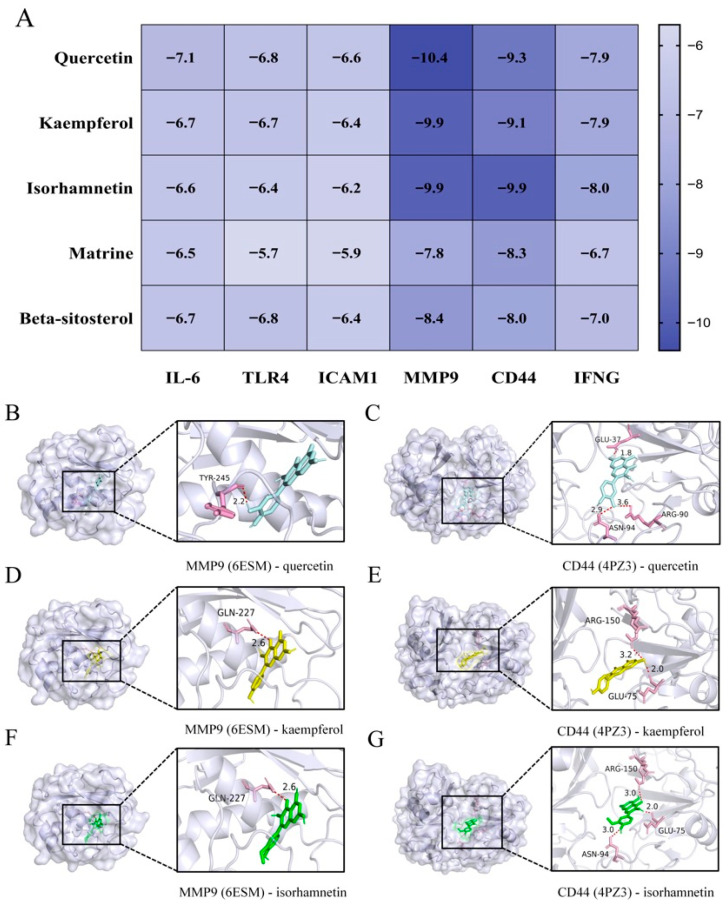
Molecular Docking Analysis of Active Compounds in **CCL**. (**A**) The heat map visualizes the binding energy of the docking of 5 core components and 6 core protein molecules. (**B**,**C**) Visualization of quercetin docking with MMP9 and CD44 molecules. (**D**,**E**) Visualization of docking of kaempferol with MMP9 and CD44 molecules. (**F**,**G**) Visualization of isorhamnetin docking with MMP9 and CD44 molecules.

To validate the interaction between Cuscutae Semen-derived bioactive compounds and the key inflammatory targets involved in ccRCC, molecular docking analysis was performed. The binding affinities between five representative compounds (quercetin, kaempferol, isorhamnetin, matrine, and β-sitosterol) and six hub proteins (IL-6, TLR4, ICAM1, MMP9, CD44, and IFNG) were evaluated using Autodock Vina. The heatmap summarized the docking scores across compound–target pairs, showing that quercetin displayed the strongest binding affinity with MMP9 (−10.4 kcal/mol), followed by CD44 (−9.3 kcal/mol) and IFNG (−7.9 kcal/mol) (Figure 2A). Other compounds, including kaempferol and isorhamnetin, also exhibited favorable binding energies, particularly with MMP9 and CD44, indicating their potential to modulate inflammation-related signaling in ccRCC. To further elucidate the binding mechanisms, molecular docking conformations of selected compound–target pairs were visualized. Quercetin was found to form hydrogen bonds with TYR-245 of MMP9 (2.2 Å), suggesting a stable interaction (Figure 2B). It also interacted with CD44 through hydrogen bonds with GLU-37 and ARG-90, with distances ranging from 2.0 to 3.6 Å (Figure 2C). Similarly, kaempferol and isorhamnetin formed strong hydrogen bonds with GLN-227 in MMP9 (2.6 and 2.7 Å, respectively) (Figure 2D–F) while also forming multiple bonds with key residues (e.g., ARG-150, ASN-94, GLU-75) in CD44 (Figure 2E–G). Overall, these docking results suggest that the active ingredients of Cuscutae Semen may exert anti-inflammatory effects in ccRCC by directly binding to core inflammatory mediators, especially MMP9 and CD44, supporting their potential pharmacological relevance in regulating tumor inflammation.

### 3.5. Anti-Tumor Effect of Quercetin Is Accompanied by a Reduction of MMP9 Expression in 786-O Cells

**Figure 3:**
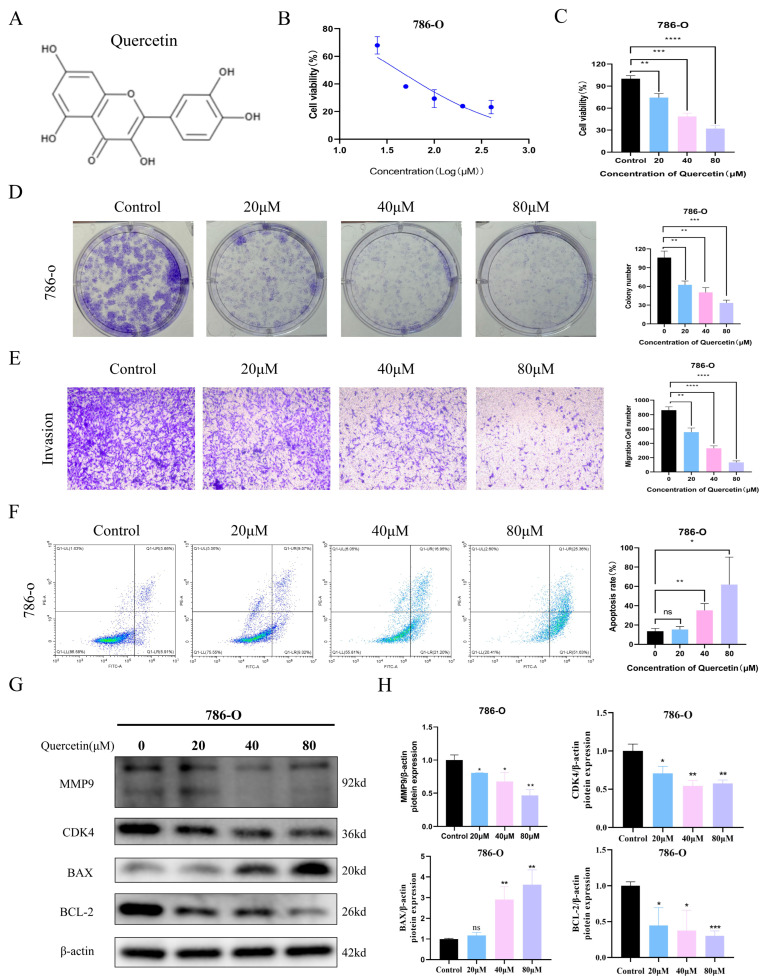
Anti-tumor effect of quercetin is accompanied by a reduction in MMP9 expression in 786-O cells. (**A**) The chemical structure of quercetin was downloaded from the TCMID database. (**B**,**C**) The MTS assay was used to detect the activity of 786-O cells treated with quercetin at different concentrations (0, 10, 20, 40, 80, 160 μM) for 48 h. (**D**) Clonal formation assay to observe the number of clones of 20, 40, 80 μM quercetin treated cells. (**E**) Transwell assay was used to detect the number of cells invaded by quercetin treated with 20, 40, 80 μM. (**F**) Flow cytometry was used to detect the change of apoptosis ratio of 20, 40, 80 μM quercetin treated cells. (**G**,**H**) Western blot analysis of MMP9, CDK4, BAX, and BCL2 expression levels in 20, 40, 80 μM quercetin treated cells. ns *p* > 0.05, * *p* < 0.05, ** *p* < 0.01, *** *p* < 0.001, **** *p* < 0.0001. The experiments were all repeated three times.

To evaluate the anti-tumor effect of **CCL**, we investigated the impact of its main active compound, quercetin, on 786-O renal carcinoma cells. A series of assays were performed to assess changes in cell viability, invasion, and apoptosis. As shown in Figure 3A, quercetin’s chemical structure was identified. Cell viability assays revealed that quercetin significantly suppressed 786-O cell viability in a dose-dependent manner, with the most substantial reduction observed at 80 μM (Figure 3B–D). The effects of the other four compounds of **CCL**, namely kaempferol, isorhamnetin, matrine, and β-sitosterol, on the 786-O cells, along with their respective IC50 values, are as follows: 29.43 μM, 14.88 μM, 94.1 μM, and 111.3 μM (Supplementary Figure S4). Similarly, colony formation assays demonstrated marked inhibition of clonogenic capacity upon quercetin treatment. Transwell assays further showed that quercetin effectively reduced the invasive potential of 786-O cells, particularly at higher concentrations (Figure 3E). In addition, apoptosis rates increased with rising doses of quercetin, confirming its pro-apoptotic effect (Figure 3F). To support the in vitro findings at the clinical level, we analyzed MMP9 expression and clinical relevance in the TCGA-KIRC dataset. MMP9 was significantly overexpressed in ccRCC tumor tissues compared to normal counterparts (Supplementary Figure S3A,B), with expression increasing across tumor stages (Supplementary Figure S3C). ROC analysis indicated that MMP9 has high diagnostic potential (AUC = 0.873; Supplementary Figure S3D). Moreover, elevated MMP9 expression was correlated with poor overall survival (OS), disease-specific survival (DSS), and progression-free interval (PFI) (Supplementary Figure S3E–H), highlighting its prognostic value.

### 3.6. Quercetin Treatment Suppressed Tumor Growth Accompanied by Decreased MMP9 Expression in the ccRCC Tumor Tissues

**Figure 4:**
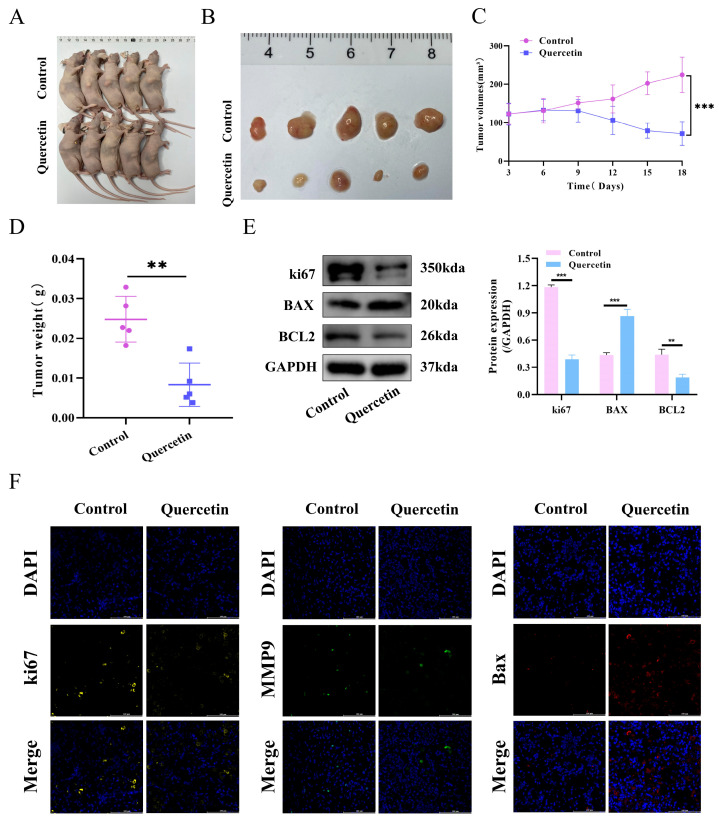
Quercetin treatment suppressed tumor growth accompanied by decreased MMP9 expression in the ccRCC tumor tissues. Mouse xenotransplantation model was established to observe the effects of quercetin treatment on tumor volume (**A**,**B**), tumor growth curve (**C**), and tumor weight (**D**) at 18 days. (**E**) The protein expression levels of Ki67, BAX, and BCL2 in quercetin-treated and untreated mouse tumor tissues were detected by WB analysis. (**F**) The expressions of ki67, MMP9 and Bax in tumor tissues of the quercetin group and control group were detected by mIHC. Scale = 100 μm. ** *p* < 0.01; *** *p* < 0.001.

A mouse xenograft model was developed to investigate the in vivo effects of quercetin on ccRCC. The control and quercetin-treated groups were compared for tumor growth and size over an 18-day period. Figure 4A,B illustrates a significant reduction in tumor size in the quercetin-treated group relative to the control group. The quercetin group exhibited a significant reduction in tumor volumes compared to the control group, as illustrated in Figure 4C, with a notable decrease observed throughout the treatment period (*p* < 0.05). Figure 4D illustrates that tumors in the quercetin group were significantly lighter than those in the control group, confirming quercetin’s efficacy in suppressing tumor growth. Western blot analysis revealed that quercetin treatment significantly downregulated the protein expression of Ki67 and BCL2 while upregulating BAX expression in the xenograft mouse tissues (Figure 4E). mIHC analysis of tumor masses revealed that quercetin treatment decreased ki67 and MMP9 expression while increasing Bax expression relative to the control group (Figure 4F).

### 3.7. MMP9 Expression Is Positively Associated with Macrophage Infiltration and M2 Polarization

**Figure 5:**
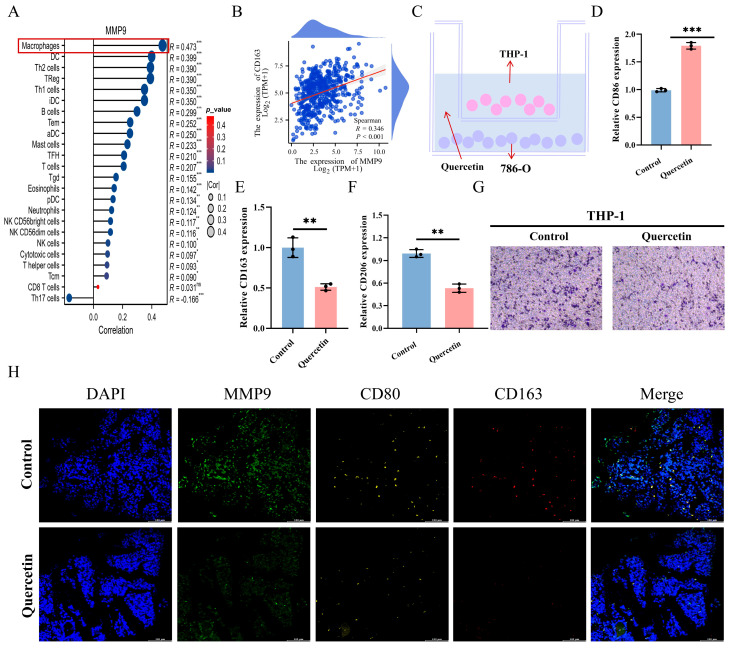
MMP9 expression positively correlated with macrophage infiltration and M2 polarization. (**A**) Correlation analysis of MMP9 expression and immune cell infiltration in TCGA database. (**B**) Correlation analysis of expression of MMP9 and M2 macrophage marker CD163 in the TCGA database. (**C**) Macrophages (THP-1) were immunocultured with 786-O cells. (**D**–**F**) mRNA expression of CD86, CD163, and CD206 in M1 macrophages treated with quercetin was detected by RT-qPCR. (**G**) Transwell assay was used to detect the invasion of co-cultured macrophages into tumor cells. (**H**) In vivo, the expression of ki67, MMP9, and Bax in quercetin-treated tumor was detected by mIHC staining after removing mouse tumor sections. ^ns^ *p* > 0.05, * *p* < 0.05, ** *p* < 0.01, *** *p* < 0.001.

A correlation analysis was performed to investigate the association between MMP9 expression and immune cell infiltration. Figure 5A demonstrates a positive correlation between MMP9 expression levels and the infiltration of various immune cells, such as macrophages. This indicates that these genes might influence the tumor microenvironment and immune response in ccRCC. Moreover, significant positive correlations were observed between the expression of MMP9 (Figure 5B), suggesting that MMP9 may regulate macrophage polarization and contribute to tumor immune escape mechanisms. Finally, Figure 5C shows the experimental setup for the co-culture of macrophages (THP-1) with 786-O cells, demonstrating the potential impact of these genes on immune cell activation. Analysis of co-cultured cell supernatants revealed a reduction in CD163 and CD206 content following quercetin treatment, indicating a decrease in M2 macrophage polarization. This suggests that quercetin may inhibit the M2 polarization process (Figure 5D). In addition, by staining the cells, we found that quercetin-treated macrophage THP-1 recruited to 786-O cells, further indicating that quercetin can promote the recruitment process of macrophages to tumor cells, and thus achieve anti-tumor effects (Figure 5E). Finally, Figure 5F shows mIHC staining for MMP9, CD80, and CD163 in tumor tissues, further confirming the inhibition of these markers in the quercetin-treated group.

### 3.8. The FSL-1 TFA Reversed the Suppressive Effect of Quercetin on the Polarization of M2-Type THP-1 Cells

**Figure 6:**
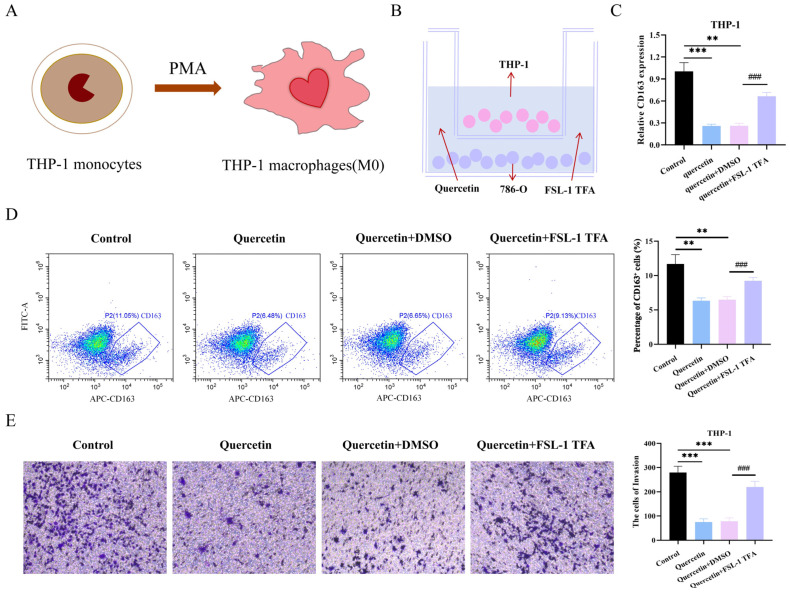
The FSL-1 TFA reversed the suppressive effect of quercetin on the polarization of M2-type THP-1 cells. (**A**) The polarization of THP-1 cells from an undifferentiated state to M0 macrophages via PMA. (**B**) M0-type THP-1 cells and 786-O cells were co-cultured in a culture medium containing quercetin and the FSL-1 TFA. (**C**) The mRNA level of CD163 in THP-1 cells after co-culture with 786-O cells was determined by RT-qPCR. (**D**) Flow cytometric analysis was used to determine the proportion of CD163-positive cells following a 24 h co-culture of M0-type THP-1 and 786-O cells (1:1 ratio) in a medium supplemented with quercetin and the FSL-1 TFA. (**E**) The invasive capacity of M0-type THP-1 cells after a 24 h co-culture with 786-O cells (1:1 ratio) in medium containing quercetin and the FSL-1 TFA was determined by the Transwell assay, with THP-1 cells seeded in the upper chamber and 786-O cells in the lower chamber. Data are presented as the mean ± SD from three independent experiments performed in triplicate. ** *p* < 0.01, *** *p* < 0.001 versus the Control group; ### *p* < 0.001 versus the quercetin + DMSO group.

To further investigate the mechanism of quercetin in ccRCC immunotherapy, we first polarized undifferentiated THP-1 cells into the M0 phenotype using PMA (Figure 6A). Subsequently, M0-type THP-1 cells and 786-O cells were co-cultured in a Transwell system for 24 h in a medium supplemented with quercetin and the FSL-1 TFA (Figure 6B). The RT-qPCR results revealed that the mRNA expression of CD163 was significantly increased in the quercetin + FSL-1 TFA group compared to the quercetin + DMSO group (*p* < 0.01, Figure 6C). Flow cytometric analysis showed that the proportion of CD163-positive cells decreased in the quercetin-treated group compared to the Control group. Conversely, the proportion of CD163-positive cells was increased in the quercetin + FSL-1 TFA group compared to the quercetin + DMSO group (*p* < 0.01, Figure 6D). More importantly, the Transwell assay results demonstrated that quercetin inhibited the invasive capacity of THP-1 cells after co-culture, and this suppressive effect was reversed by the addition of FSL-1 TFA (*p* < 0.01, Figure 6E).

### 3.9. The FSL-1 TFA Reversed the Abrogation of Quercetin-Mediated Suppression of ccRCC Malignant Progression

**Figure 7:**
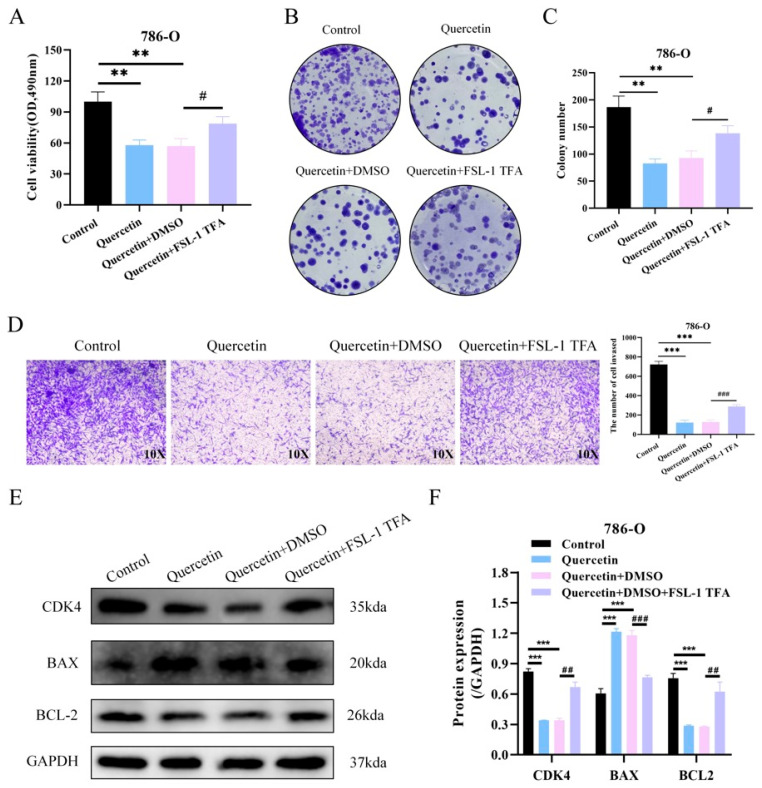
Abrogation of quercetin-mediated suppression of ccRCC malignant progression by FSL-1 TFA. (**A**) Cell viability of 786-O cells following treatment with quercetin in combination with FSL-1 TFA was assessed by the MTS assay. (**B**) Proliferation ability of 786-O cells following treatment with quercetin in combination with FSL-1 TFA was assessed by the colony formation assay. (**C**,**D**) Invasive ability of 786-O cells following treatment with quercetin in combination with FSL-1 TFA was assessed by the Transwell assay. (**E**,**F**) Western blot analysis of CDK4, BAX, and BCL2 expression in 786-O cells following 48-h combined treatment with quercetin and FSL-1 TFA. ** *p* < 0.01, *** *p* < 0.001. ## *p* < 0.01, ### *p* < 0.001. # Compared to the quercetin + DMSO group.

Treatment with quercetin significantly reduced the cell viability compared with the control group, as measured by the CCK-8 assay. However, this inhibitory effect was partially reversed upon co-treatment with the FSL-1 TFA (Figure 7A). Colony formation assays further demonstrated that quercetin markedly suppressed the clonogenic potential of the 786-O cells, while FSL-1 treatment restored this ability to some extent (Figure 7B,C). Similarly, Transwell invasion assays revealed that quercetin significantly inhibited cell invasiveness, and this suppression was notably alleviated by FSL-1 (Figure 7D). Quantification of invasive cells confirmed the statistical significance of these findings. Western blot analysis showed that quercetin treatment led to a significant decrease in CDK4 and BCL-2 protein levels, accompanied by an upregulation of the pro-apoptotic protein BAX compared to the control. Co-treatment with FSL-1 reversed these trends, indicating that TLR2 activation counteracts the effects of quercetin on cell cycle progression and apoptosis regulation (Figure 7E,F).

## 4. Discussion

RCC, particularly ccRCC, is a highly aggressive malignancy with limited effective treatment options, especially in advanced stages [28,29,30,31]. Although targeted therapies such as tyrosine kinase inhibitors and immune checkpoint inhibitors have improved patient outcomes, resistance and immune evasion remain major challenges [32,33,34]. TCM has attracted increasing attention as a complementary approach to cancer therapy due to its multi-target and multi-pathway mechanisms [35,36,37,38]. We explored the therapeutic effects of **CCL** on ccRCC through network pharmacology, molecular docking, and experimental validation in vitro and in vivo. Our findings strongly indicate that CCL exerts anti-tumor effects by modulating the immune system, inhibiting tumor proliferation and invasion, and regulating the PI3K/AKT signaling pathway. Network pharmacology analysis revealed 251 shared targets between **CCL** and ccRCC, highlighting hub genes like MMP9, IL6, FN1, and TLR4 as pivotal in ccRCC progression. Functional enrichment analysis revealed that these targets are significantly involved in immune responses, inflammation, and hypoxia adaptation, which are critical factors contributing to ccRCC development. The PI3K/AKT signaling pathway’s role as a key regulatory mechanism indicates that **CCL** may influence tumor cell survival, immune evasion, and microenvironment interactions. These findings align with the growing body of evidence indicating that PI3K/AKT signaling plays a pivotal role in ccRCC progression and drug resistance, making it a promising therapeutic target. Molecular docking further validated the potential of CCL as a ccRCC therapy, demonstrating strong binding affinities between its active compounds and key target proteins. Among the bioactive components of CCL, quercetin exhibited the highest binding affinity to MMP9, a protein closely linked to tumor invasion and metastasis. MMP9 is well-known for its role in degrading the ECM and facilitating the invasion of RCC cells, suggesting that CCL may inhibit tumor dissemination. Accumulating evidence has established MMP9 as a critical player in ccRCC aggressiveness. Multiple studies have demonstrated that MMP9 is significantly overexpressed in ccRCC tissues compared to adjacent normal tissues at both the mRNA and protein levels. This upregulation is associated with poor prognosis, as high MMP9 expression correlates with reduced OS in ccRCC patients. Mechanistically, MMP9 expression in ccRCC may be driven by activation of the MAPK/ERK signaling pathway, which subsequently upregulates transcription factors such as ETS1. As a matrix metallopeptidase, MMP9 plays a fundamental role in ECM degradation, particularly targeting type IV collagen—a major component of the basement membrane. This proteolytic activity facilitates tumor cell invasion and metastasis by disrupting the physical barrier and remodeling the tumor microenvironment. Recent studies have also revealed that neutrophil extracellular traps (NETs) can promote ccRCC progression via the TGFβ1–pSmad2–MMP9 signaling axis, further highlighting MMP9’s central role in ECM dynamics and metastasis.

Our in vitro experiments provide direct evidence that quercetin significantly reduces ccRCC cell viability, inhibits proliferation, and suppresses invasion. The CCK-8 assay indicated that quercetin treatment reduced cell viability in a dose-dependent manner, and both colony formation and invasion assays confirmed its inhibitory effects on ccRCC cell proliferation and migration. Interestingly, flow cytometry analysis revealed that while quercetin induces apoptosis, its primary anti-tumor effect appears to be through the inhibition of cell proliferation rather than apoptosis induction. This observation is consistent with previous reports that quercetin predominantly affects the cell cycle and tumor microenvironment rather than directly triggering apoptosis in certain cancer models. The in vivo xenograft mouse model provided additional validation of CCL’s anti-tumor activity. Quercetin-treated mice showed significantly reduced tumor volumes and weights compared to the control group. Histological examination indicated increased necrosis and decreased cellular density in tumor tissues, supporting the hypothesis that CCL inhibits tumor growth. Quercetin effectively inhibits ccRCC progression in both in vivo and in vitro studies. The findings demonstrate that quercetin significantly reduces MMP9 expression in both 786-O cells and tumor tissues of ccRCC animal models. The findings, supported by network pharmacology and molecular docking analyses, suggest that quercetin may inhibit ccRCC development by targeting MMP9.

A significant outcome of our study is the impact of CCL on the tumor immune microenvironment (TIME). ccRCC is characterized by an immunosuppressive environment with tumor-associated macrophages (TAMs) that facilitate tumor growth and immune evasion. Importantly, MMP9 is intimately involved in tumor immune microenvironment modulation, particularly in macrophage polarization. Emerging evidence indicates that MMP9 serves as a downstream target of the NF-κB pathway, which critically regulates M2 macrophage polarization. A recent study demonstrated that monotropein suppresses ccRCC progression and reduces M2 macrophage polarization by inhibiting the NF-κB pathway and subsequently downregulating MMP9 expression. This suggests that MMP9 not only functions as a protease but also as a mediator of immunosuppressive tumor microenvironment formation. Furthermore, MMP9 has been identified as a gene that can stratify ccRCC patients from healthy controls based on circulating lymphocyte expression profiles, underscoring its potential as a non-invasive diagnostic marker. Our analysis indicates a positive correlation between MMP9 expression and macrophage infiltration in ccRCC tissues, implying its involvement in recruiting or activating pro-tumoral immune cells. Additionally, the strong correlations between MMP9 and CD163 indicate that CCL may exert its effects by shifting macrophage polarization from an immunosuppressive M2 phenotype toward an anti-tumor immune response. Immunohistochemical and multiplex immunofluorescence staining further confirmed that quercetin treatment downregulates MMP9, CD80, and CD163 expression in xenograft tumor tissues, suggesting that CCL can alter the immune landscape in ccRCC. More importantly, to explore the immunotherapeutic mechanism of quercetin in ccRCC, we conducted a reversal experiment using the MMP9 activator FSL-1 TFA and found that FSL-1 TFA could reverse the inhibition of quercetin on M2-type macrophages, further indicating that MMP9 might be an important target for the immune effect of quercetin against ccRCC, laying a theoretical foundation for quercetin in the immunotherapy of ccRCC.

There are a number of limitations to this study, despite the encouraging results. Although the present study demonstrates that quercetin downregulates MMP9 expression and that FSL-1 partially reverses its biological effects, the current results do not provide definitive genetic evidence that MMP9 directly mediates these effects. Future studies using gene silencing or CRISPR-Cas9/overexpression approaches could help dissect the specific contributions of individual targets such as MMP9 and PI3K/AKT signaling in the therapeutic effects of quercetin. Future studies incorporating gelatin zymography will be necessary to determine whether quercetin directly affects the enzymatic activity of MMP9. Second, because the xenograft experiments were performed in BALB/c nude mice lacking functional T cells, the immune-related findings should be interpreted cautiously. Future studies using immunocompetent mouse models will be necessary to fully evaluate the effects of quercetin on the tumor immune microenvironment. Third, although our study indicates that CCL affects the tumor immune microenvironment, comprehensive immune profiling through flow cytometry or single-cell RNA sequencing (scRNA-Seq) is necessary to thoroughly understand its immunomodulatory effects. Future studies using immunocompetent models will be required to confirm immune microenvironment regulation, and examining mitochondrial apoptosis pathways, including caspase-3 activation, PARP cleavage, mitochondrial membrane potential (JC-1 staining), and cytochrome-c release would provide deeper insight into the apoptotic mechanisms of quercetin.

## 5. Conclusions

This study demonstrates that quercetin exhibits anti-tumor effects in ccRCC by modulating immune responses, thereby inhibiting tumor proliferation and invasion. Using network pharmacology and experimental validation, we identified MMP9 as a key molecular target that mediated M2 macrophage polarization to inhibit the malignant progression of ccRCC. Our research underscores the promise of CCL as a complementary therapy for ccRCC, paving the way for future investigations into its clinical applications.

## Figures and Tables

**Table 1:** The screening information for active medicinal components.

Mol ID	Molecule Name	OB (%)	DL
MOL001558	Sesamin	56.55	0.83
MOL000184	NSC63551	39.25	0.76
MOL000354	Isorhamnetin	49.6	0.31
MOL000358	Beta-sitosterol	36.91	0.75
MOL000422	Kaempferol	41.88	0.24
MOL005043	Cmpest-5-en-3beta-ol	37.58	0.71
MOL005440	Isofucosterol	43.78	0.76
MOL005944	Matrine	63.77	0.25
MOL006649	Sophranol	55.42	0.28
MOL000953	CLR	37.87	0.68
MOL000098	Quercetin	46.43	0.28

## Data Availability

All authors agree that the data in the manuscript should be published, and all data in the text should be available from the corresponding author.

## Supplementary Materials

The following supporting information can be downloaded at: https://www.mdpi.com/article/10.3390/biomedicines14040904/s1, Figure S1: Functional Enrichment and Pathway Analysis of Key Targets.; Figure S2: Network and Molecular Interactions of Key Genes in ccRCC; Figure S3: Expression and Correlation Analysis of Key Genes in ccRCC; Figure S4: The effects of the other four CCL compounds (namely kaempferol, isorhamnetin, matrine and β-sitosterol) on the activity of 786-O cells.

## Abbreviations

ccRCC	Clear cell renal cell carcinoma
FSL-1 TFA	Toll-like receptor 2/6 agonist
CTD	Comparative Toxicogenomics Database
DL	Drug-likeness
DEGs	Differentially expressed genes
ECL	Enhanced chemiluminescence
EPC	Edge Percolated Component
FBS	Fetal bovine serum
GEO	Gene Expression Omnibus
GO	Gene Ontology
GEMMs	Genetically engineered mouse models
H&E staining	Hematoxylin and eosin
ICIs	Immune checkpoint inhibitors
KEGG	Kyoto Encyclopedia of Genes and Genomes
MCC	Maximal Clique Centrality
mIHC	Multiplex immunohistochemistry
OB	Oral bioavailability
PPI	Protein–protein interaction
PDB	Protein Data Bank
PDX	Patient-derived xenografts
scRNA-seq	single-cell RNA sequencing
TCM	Traditional Chinese medicine
TIME	Tumor immune microenvironment
TAMs	Tumor-associated macrophages
TKIs	Tyrosine kinase inhibitors
