# Supplementary material for: Integrated Network Pharmacology, Molecular Docking and Experimental Validation Reveal That Quercetin Suppresses Clear Cell Renal Cell Carcinoma via MMP9-Associated Macrophage Polarization
Source: Biomedicines. 2026 Apr 16;14(4):904. doi: 10.3390/biomedicines14040904 (PMC13113011; doi:10.3390/biomedicines14040904)
Supplement: Supplementary file 1 [file biomedicines-14-00904-s001.zip › biomedicines-4074941-supplementary.pdf]

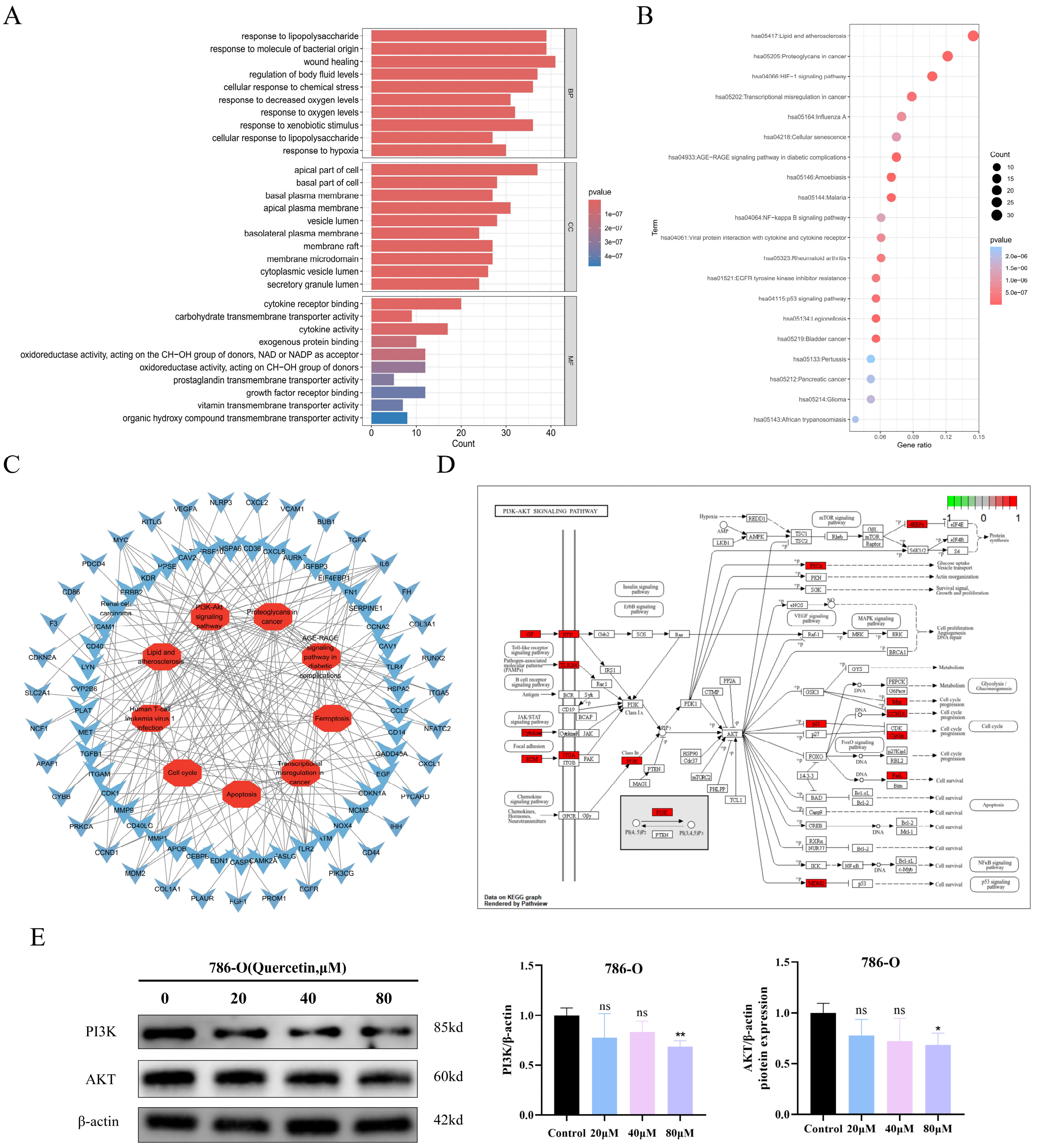

Supplementary Figure S1. Functional Enrichment and Pathway Analysis of Key Targets. (A)GO enrichment analysis visualizes the biological functions of BP, CC, MF in the top 10 entries of potential CCL and ccRCC targets. (B) KEGG enrichment analysis visualizes the signaling pathways of the top 20 items of CCL and ccRCC potential targets. (C) Cytoscape Visualizes the "target-pathway" network. (D) R software visualizes the mechanism flow of PI3K/AKT signaling pathway.

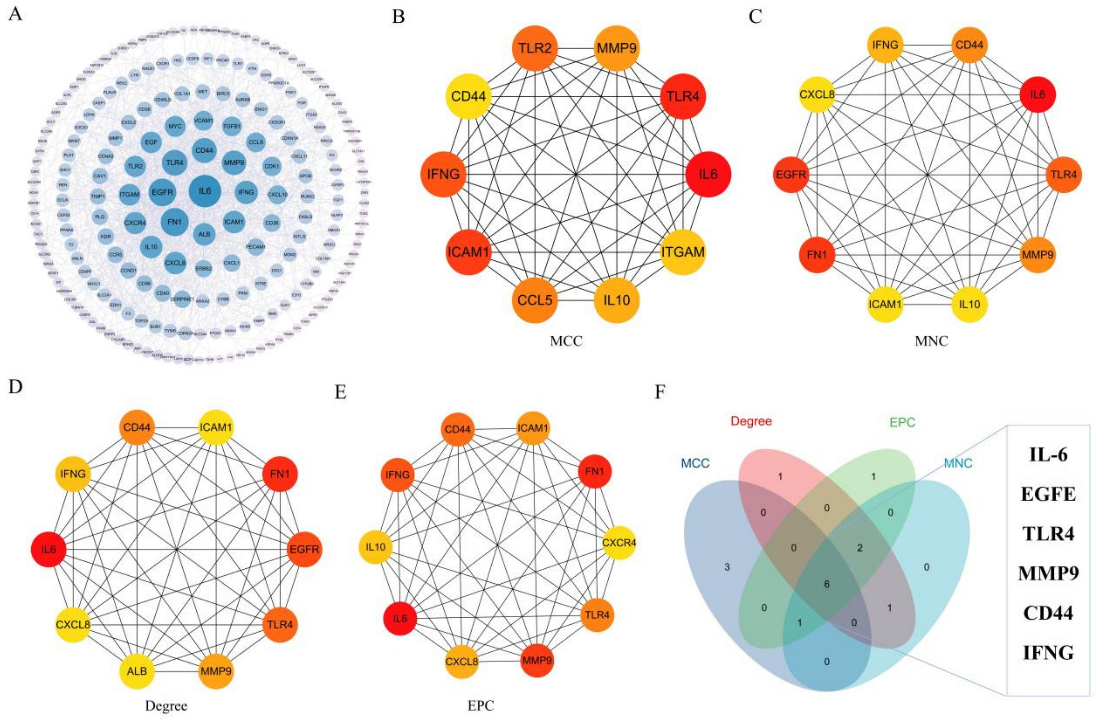

Supplementary Figure S2. Network and Molecular Interactions of Key Genes in ccRCC. (A) Cytoscape software builds the PPI network at the common target of CCL and ccRCC. The top 10 core target networks are constructed by MCC (B), MNC (C), Degree (D) and EPC (E) algorithms in Cytoscape software. (F) VN diagram visualizes the core targets obtained by MCC, MNC, Degree and EPC algorithms.

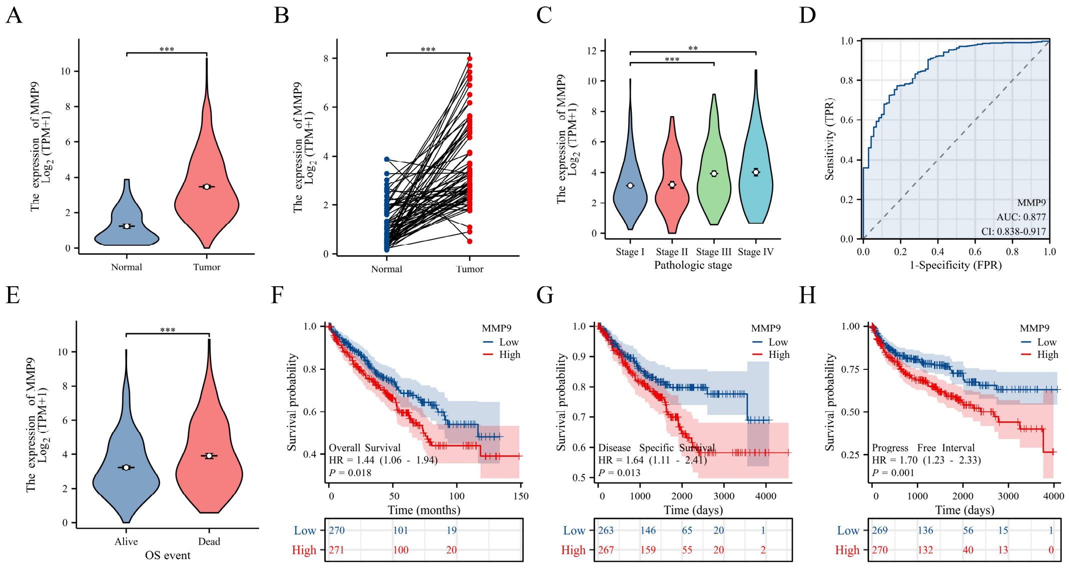

Supplementary Figure S3. Expression and Correlation Analysis of Key Genes in ccRCC. (A, B) Expression levels of MMP9 in unpaired and paired ccRCC samples from the TCGA database. (C) Different pathological grades of ccRCC. (D)The area under the ROC curve of MMP9. (E) Analysis of the expression level of MMP9 and its diagnostic significance for OS. Survival analysis of MMP9 and OS (F), DSS(G), and PFI(H) in high and low groups.

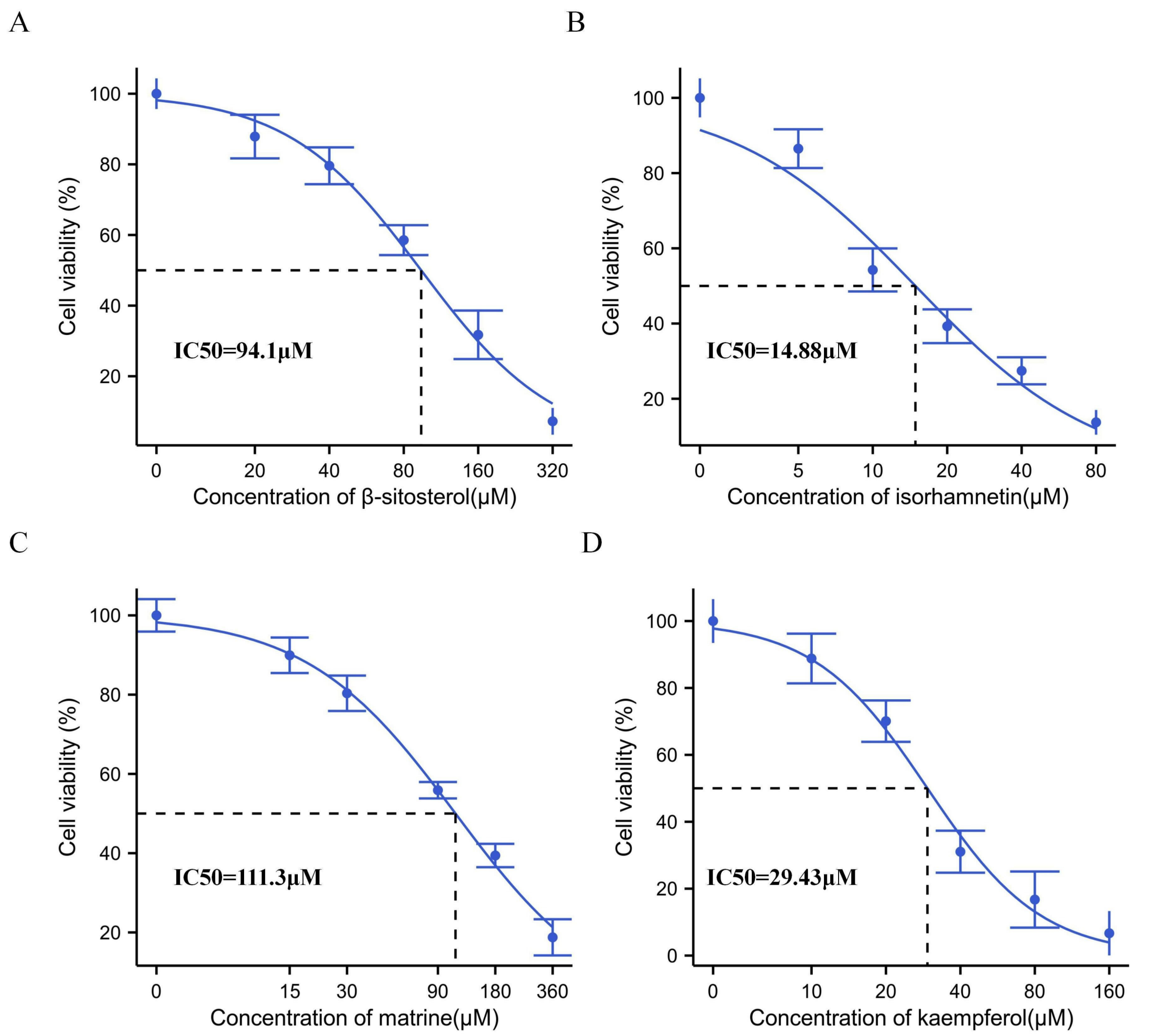

Supplementary Figure S4. The effects of the other four CCL compounds (namely kaempferol, isorhamnetin, matrine and β-sitosterol) on the activity of 786-O cells.
